# Integrated analysis of multiple receptor tyrosine kinases identifies Axl as a therapeutic target and mediator of resistance to sorafenib in hepatocellular carcinoma

**DOI:** 10.1038/s41416-018-0373-6

**Published:** 2019-02-15

**Authors:** David J. Pinato, Matthew W. Brown, Sebastian Trousil, Eric O. Aboagye, Jamie Beaumont, Hua Zhang, Helen M. Coley, Francesco A. Mauri, Rohini Sharma

**Affiliations:** 1 Department of Surgery and Cancer, Imperial College London, Hammersmith Hospital Campus, Du Cane Road, London, W12 0NN UK; 2 Department of Oncology, New York University, Laura and Isaac Perlmutter Cancer Center, New York, NY USA; 3 Faculty of Health and Medical Sciences, University of Surrey, Guildford, Surrey UK; 4 Department of Histopathology, Imperial College London, Hammersmith Hospital Campus, Du Cane Road, London, W12 0HS UK

**Keywords:** Hepatocellular carcinoma, Target identification

## Abstract

**Background:**

Aberrant activation of Axl is implicated in the progression of hepatocellular carcinoma (HCC). We explored the biologic significance and preclinical efficacy of Axl inhibition as a therapeutic strategy in sorafenib-naive and resistant HCC.

**Methods:**

We evaluated Axl expression in sorafenib-naive and resistant (SR) clones of epithelial (HuH7) and mesenchymal origin (SKHep-1) using antibody arrays and confirmed tissue expression. We tested the effect of Axl inhibition with RNA-interference and pharmacologically with R428 on a number of phenotypic assays.

**Results:**

Axl mRNA overexpression in cell lines (*n* = 28) and RNA-seq tissue datasets (*n* = 373) correlated with epithelial-to-mesenchymal transition (EMT). Axl was overexpressed in HCC compared to cirrhosis and normal liver. We confirmed sorafenib resistance to be associated with EMT and enhanced motility in both HuH7-SR and SKHep-1-SR cells documenting a 4-fold increase in Axl phosphorylation as an adaptive feature of chronic sorafenib treatment in SKHep-1-SR cells. Axl inhibition reduced motility and enhanced sensitivity to sorafenib in SKHep-1SR cells. In patients treated with sorafenib (*n* = 40), circulating Axl levels correlated with shorter survival.

**Conclusions:**

Suppression of Axl-dependent signalling influences the transformed phenotype in HCC cells and contributes to adaptive resistance to sorafenib, providing a pre-clinical rationale for the development of Axl inhibitors as a measure to overcome sorafenib resistance.

## Introduction

The majority of patients with a new diagnosis of hepatocellular carcinoma (HCC), the third cause of cancer-related death, present with incurable disease.^1^ Whilst loco-regional therapies may represent an option for those with liver-confined tumours,^2^ the disease will inevitably progress after initial treatment, with metastatic spread reducing the chances of long-term survival.^3^

Patients with advanced stage disease have an expected overall survival (OS) of 12 months.^4^ Sorafenib, a multi-targeted tyrosine kinase inhibitor (TKI) of Raf, vascular endothelial growth factor (VEGF) and platelet-derived growth factor (PDGF) improve survival by approximately 45% over placebo.^5,6^ However, sorafenib, only delays median time to radiologic progression by an average of 3 months,^5^ and advanced stage patients have been the focus of intense research efforts to identify novel treatment strategies to be administered either as a more efficacious frontline alternative to sorafenib or as second-line therapy following the emergence of resistance or intolerance to sorafenib.^7^ With the recent exception of regorafenib in the second-line setting^8^ and lenvatinib,^9^ all compounds tested beyond phase II have been proven ineffective, resulting in late-stage failures in the drug development process.^10^ Unlike other solid tumours where genomic stratification has refined treatment allocation based on the likelihood of response to TKIs; a clear, treatment-guiding molecular classification has not yet emerged in HCC, with inherent negative implications in drug development and trial design.^11^ The acute demand for new systemic therapies has therefore made the qualification of novel therapeutically meaningful signalling pathways a priority in HCC.^12^

The receptor tyrosine kinase (RTK) Axl, a member of the TAM subfamily that also includes Mer and Tyro3, has been identified as a transforming oncogene with its activation being implicated in several biologic responses including cell proliferation, survival and motility across a wide range of malignancies.^13^ Axl binds preferentially to a soluble ligand, growth arrest signal 6 (Gas-6), and mediates its intracellular functions via activation of phosphatidyl-inositol-3 kinase (PI3K)/Akt^14^ and, to a lesser extent, ERK-p38 mitogen-activated protein kinase (MAPK).^15^ Previous mechanistic evidence has demonstrated the relevance of Axl in the progression of HCC^16^ identifying Axl as a downstream regulator of the Hippo signalling pathway, a key regulator of tissue development, whose disruption is implicated in tumour cell migration, invasion and proliferation through MAPK activation. Axl is also implicated as a molecular determinant of tumour invasiveness in the context of epithelial to mesenchymal transition (EMT),^17,18^ a coordinated gene expression program largely governed by the transcription factors Slug, Snail and Twist that endow tumour cells with enhanced motility, invasive and metastatic capacity through E-cadherin repression and Vimentin overexpression.^19^ Axl is an essential downstream regulator of EMT and is required for the metastatic spread of a number of malignancies.^20,21^ More recently, Axl has been implicated in the development of acquired resistance to a number of molecularly targeted therapies across a wide range of malignancies, suggesting EMT and Axl as pivotal features characterising adaptive resistance to anti-angiogenics and multi-targeted kinase inhibitors.^22^

Whilst a number of compensatory pathways, including EMT,^23^ have been highlighted as putative mechanistic drivers of sorafenib resistance, the role of Axl has not been addressed by the previous studies.^24^ We investigated the biologic relevance of Axl in the progression of HCC and in the acquisition of adaptive resistance to sorafenib. Using in-vitro models, we subsequently evaluated whether its inhibition could represent a potential therapeutic strategy in the systemic treatment of HCC.

## Materials and methods

### Cell cultures

The human HCC cell lines PLC/PRF/5, Hep3B, HuH7, SKHep-1, SNU-449, SNU-387 were obtained from American Type Culture Collection (ATCC, Manassas, VA, USA). All cell lines were cultured according to standard procedures using cell-specific culture medium in the presence of 10% fetal calf serum (FCS). HuH7-SR and SKHep-1-SR cells were generated by growing parental cells under increasing concentrations of sorafenib (up to 10 μM). Surviving cells were passaged weekly and a stable growth rate at the maintenance concentration of 6 μM was achieved after 6 months.

### Immunoblotting

Immunoblotting was performed as described by our group before^25^ following cell lysis in RIPA buffer (Invitrogen, Paisley, UK) supplemented with protease and phosphatase inhibitor cocktails (Sigma, St. Louis, MO, USA). The full list of antibodies utilised can be found in Supplementary Materials and Methods.

### Drugs

Sorafenib was purchased from Selleckchem (Houston, TX, USA) and R428 was kindly provided by Dr. Sacha Holland (Rigel Therapeutics, San Francisco, CA, USA).

### Target knockdown by RNA interference

Axl expression was downregulated using a mixture of three individual siRNA (ON-TARGET*plus* SMARTpool, Dharmacon, Chicago, IL, USA) and tested with control non-target sequences (siGENOME non-targeting shRNA pool) as described before.^25^

### Growth-inhibition assay

Drug concentrations capable of inhibiting 50% of cell growth (GI 50) were extrapolated using the sulphorhodamine-B assay.^26^ Drug treatment was continued for 72 h starting on day 2 from cell seeding. Combination of sorafenib with R428 was evaluated using the Combination Index method^27^ (Supplementary Methods) on CompuSyn software 1.0 (Combosyn Inc., Paramus, NJ, USA).

### ^18^FDG cell uptake assay

^18^F-FDG uptake studies were carried out as previously described with modifications.^28^ In brief, SKHep-1 cells were seeded in 12-well plates 48 h before uptake assay and treated for 6 or 24 h with indicated doses of R428. Cells were incubated with 0.74 MBq/mL ^18^F-FDG (PETNET, Nottingham, UK) for 60 min. Cells were trypsinised, washed three times with PBS and lysed in RIPA buffer. The radioactivity was counted on a Packard Cobra II gamma counter (Perkin Elmer) and radioactivity was normalised to applied radioactivity and protein content, as determined by BCA assay.

### Measurement of soluble Axl in serum

Following written, informed consent (Ethics Ref. No. 17/YH/0015) plasma samples from 40 patients with HCC were obtained before sorafenib treatment (Nexavar^®^, Bayer Schering Pharma). Concentrations of Axl were measured using a commercial sandwich ELISA kit (EHAXL, ThermoFisher Scientific, Waltham, MA, USA) according to manufacturer’s instructions.

### Cell cycle analysis

Cells were treated with R428 for 24 h then collected, fixed with ethanol and stained with propidium iodide in PBS for 3 h. Cell cycle distribution was determined using flow cytometry (FACS Canto, Becton Dickinson, Oxford, UK) and analysed using the FlowJo software (Treestar Inc., Ashland, OR, USA). In each analysis, 10.000 events were recorded.

### Migration and invasion assays

50,000 cells were seeded in 300 μL of serum-free media in 24-wells, 8.0 μm pore transwell chambers (Corning, Corning, NY, USA). Lower chambers were filled with 10% FCS medium. Drug treatment was applied to both chambers. Following 18 h incubation, membranes were fixed in pure methanol and stained with 0.4% crystal violet in 20% methanol. Non-migrated cells were removed with a cotton swab. The number of invasive cells was quantified in triplicate on 20× magnification photographs. Cell migration and invasion in response to R428 was further evaluated using real-time cell analysis (RTCA) using the xCELLigence platform (Acea Bioscience, San Diego, CA, USA) as previously described. Cell index (CI) values at landmark timepoints were analysed across experimental conditions (Supplementary Methods).^29^

### Wound healing assays

Cells were plated in 12-well tissue culture plates and maintained until 95% confluent. After overnight starvation in serum-free media, a scratch was made on the cell monolayer using a 200 μL sterile micropipette tip. Initial gap widths (0 h) and residual gap widths at 8 h were determined from photomicrographs. Cells were subjected to transfection or drug treatment prior to plating and maintained in drug-conditioned media throughout the experiment.

### Matrigel clonogenic assay

Single cell suspensions (12.500/mL) were plated on a matrigel-coated 8-well slide (Sigma Aldrich) and resuspended in full media containing 2% matrigel. Phenotypic characteristics of colonies were evaluated 7 and 14 days after treatment on 20× magnification photographs. For drug treatment with R428, media were changed every 3 days.

### Antibody arrays

We used the Pathscan RTK Antibody Array kit (7982, Cell Signaling Technology) to simultaneously evaluate 28 RTK and 11 signalling nodes in sorafenib-naive and resistant clones. Signal intensities were analysed using ScanAlyze array software (Eisen Lab Software) and normalised signal intensity values were derived as described before.^30^

### Immunohistochemistry

Expression of Axl and Gas-6 was studied by immunohistochemistry (IHC) on primary paraffin-embedded HCC specimens following pathological review of diagnostic haematoxylin and eosin sections by a certified pathologist (F.A.M.) to identify areas of tumour and surrounding cirrhosis. Ten cases of normal liver tissue obtained from hepatectomy specimens for other indications were used as controls. The primary antibodies were incubated overnight at the concentration of 1:50 for anti-Gas-6 (Cat. No. HPA008275, Sigma Aldrich), Axl (Cat. No. HPA037422, Sigma Aldrich), as previously described.^31^ Protein expression was quantified using the immuno-histoscore (IHS) method.^32^ Briefly, each specimen was scored on a semi-quantitative scale ranging from 0 to 300, with the final score resulting from the percentage of tumour cells staining positively (range 0–100) multiplied by staining intensity graded as negative, weak, moderate or strong (range 0–3). A separate IHS value was given for both areas of cirrhosis and HCC. To further explore the relationship between Axl and the metastatic progression of HCC, we constructed an isogeneic series of 12 matched primary and metastatic HCC samples obtained from 5 patients with advanced HCC (Supplementary Materials and Methods). Access to retrospective tissue specimens was granted by the Imperial College Tissue Bank (Approval No. R16005).

### The Cancer Genome Atlas (TCGA) and Cancer Cell Line Encyclopedia (CCLE) analysis

To provide further insight around the biologic significance of Axl expression, we evaluated gene expression profiles derived from the CCLE dataset of HCC cell lines (*n* = 28) and validated these in human samples using the TCGA RNASeq v2 dataset (*n* = 373). Positive and negative correlations between transcripts were sought using Pearson’s *R* scores.

### Gene set enrichment analysis (GSEA)

To characterise signalling pathways associated with Axl expression, we performed GSEA using RNA-sequencing data from the CCLE RNAseq dataset of 28 HCC cell lines as described before.^33^ Axl was used as a phenotypic label and was correlated with the validated EMT hallmark signature derived from the MSigDB database. A Benjamini–Hochberg corrected false-discovery rate *q* value of <0.25 was considered significant for gene set enrichment.

### Statistical analysis

Continuous variables were expressed as means ± standard deviation (SD) or medians ± interquartile ranges (IQR). Differences in means were assessed for statistical significance by means of Student *T*-test. Survival analyses were conducted using Kaplan–Meier statistics and Log-rank tests. For all analyses, *p* value < 0.05 (two-tailed) was taken to be significant. Statistical analyses were conducted using SPSS statistical package 20.0 (IBM Inc., Armonk, NY, USA) and GraphPad (GraphPad Software, La Jolla, CA, USA).

## Results

### Axl is overexpressed in HCC and is associated with EMT

**Fig. 1:**
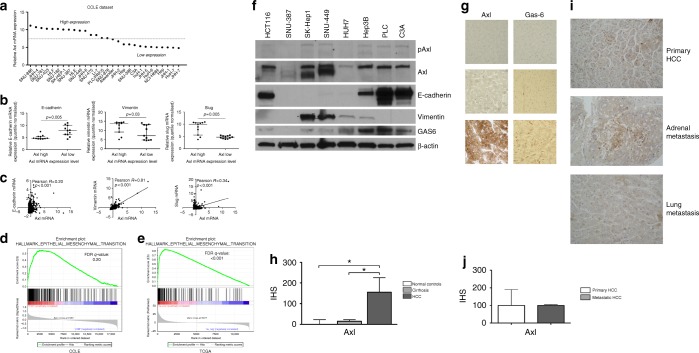
Axl overexpression is a common feature of hepatocellular carcinoma (HCC) and is associated with epithelial-to-mesenchymal transition (EMT). **a** Differential expression of Axl mRNA in a panel of 28 immortalised HCC cell line from the Cancer Cell Line Encyclopedia (CCLE) dataset confirming Axl over-expression in 13/28 cell lines. **b**, **c** The relationship between the expression of Axl and EMT-related genes including E-Cadherin, Vimentin and Slug in the CCLE (**b**) and in The Cancer Genome Atlas (TCGA) HCC RNA-seq dataset (*n* = 373). **d**, **e** GSEA on the CCLE RNA-seq dataset of 28 HCC cell lines (**d**) and TCGA dataset (**e**) confirmed enrichment of EMT-related transcripts in Axl-overexpressing cell lines (FDR *q* = 0.20 and *q* < 0.001, respectively). **f** Analysis of the expression of Axl and related total and phosphorylated protein by Western blotting in a panel of 7 HCC cell lines with colorectal cancer HCT116 cell lines serving as positive controls. **g** Representative sections of normal (*n* = 10), cirrhotic (*n* = 10) livers and HCC (*n* = 10) demonstrating Axl and Gas-6 expression by immunohistochemistry. HCC samples display a strong, membranous staining for Axl in neoplastic cells and scattered Gas-6 expression in peri-tumoural Kupffer cells. **h** The distribution of Axl expression by immunohistoscores (IHS) across normal, cirrhotic liver tissues and HCC (*n* = 10 in each group) demonstrating significant Axl overexpression in tumour samples. **i** Representative sections demonstrating Axl expression in matched primary and metastatic HCC specimens. **j** The distribution of Axl expression in primary and metastatic HCC samples (*n* = 12) derived from 5 patients

We used the CCLE database to investigate the expression of Axl and its ligand Gas-6 in a panel of 28 immortalised HCC cell lines. The median normalised Axl mRNA value was 7.6 (range 4.9–11.1), with Axl overexpression, defined as Axl mRNA normalised values above the median of the distribution, being detected in 13/28 of the studied cell lines (Fig. 1a). Axl overexpression correlated negatively with E-cadherin and positively with Slug and Vimentin mRNA expression (*p* = 0.001), consistent with EMT (Fig. 1b).

Analysis of the TCGA dataset confirmed significant linear relationship between the expression of Axl and Vimentin (Pearson *R* = 0.81, *p* < 0.0001), Slug (*R* = 0.34, *p* < 0.0001), and E-cadherin expression (*R* = 0.20, *p* < 0.001) as shown in Fig. 1c. GSEA analysis on the CCLE RNA-seq dataset inclusive of all the 28 HCC cell lines confirmed enrichment of transcripts pertaining to EMT in Axl-overexpressing cell lines (FDR *q* = 0.20, Fig. 1d), which was validated in the TCGA dataset (FDR *q* < 0.001, Fig. 1e). Median Gas-6 mRNA normalised value was 6.3 (range 5.4–10.6) and Axl/Gas-6 co-expression was confirmed in both the CCLE database (Pearson *R* = 0.70, *p* < 0.001) and the TCGA tissue dataset (Pearson *R* = 0.36, *p* < 0.0001) Supplementary Figure 1A-B.

We confirmed Axl expression by Western blot on a restricted panel of 7 HCC cell lines, using HCT-116 lysates as a positive control. Immunoblot analysis confirmed that the highest Axl expressing cell lines (SKHep-1, SNU-449) had suppressed E-cadherin expression and strong Vimentin expression, consistent with EMT activation (Fig. 1f) but no relationship with Akt phosphorylation at the Ser^473^ in untreated cell lysates (Supplementary Figure 2).

### Axl is expressed in primary and metastatic human HCC tissue samples

We assessed Axl and Gas-6 expression by IHC in archival, paraffin-embedded tissue samples of resected HCC, background cirrhosis and in normal controls (*n* = 10 in each group) (Fig. 1g, h). In primary HCC, IHS values for Axl ranged from 0 to 270 (mean 147 ± 89 SD), and 60% were categorised as high expression (defined as IHS ≥ 147). Axl expression ranged from 0 to 50 (mean 16 ± 15 SD) in matched cirrhotic tissue. Six out of 10 normal controls tissues were negative for Axl expression, with mild grade immunolabeling seen in the remaining four (range 0–90, mean 16 ± 28 SD). Gas-6 was not detected by IHC within hepatocytes in normal, cirrhotic or neoplastic tissues, being restricted to Kupffer cells.

Because Axl is involved in the metastatic progression of malignancy we evaluated Axl expression in an isogeneic collection of primary and metastatic deposits derived from advanced HCC patients, whose clinico-pathologic features are summarised in Supplementary Table 1. Axl expression ranged from 70 to 200 in primary (mean 130 ± 56 SD) and 70 to 120 (mean 93 ± 20 SD) in corresponding metastatic deposits (Fig. 1i) with uniform expression levels across primary and metastatic disease (Fig. 1j, *p* = 0.36).

### Pharmacologic inhibition of Axl is cytotoxic and modulates the transformed phenotype in HCC cell lines

**Fig. 2:**
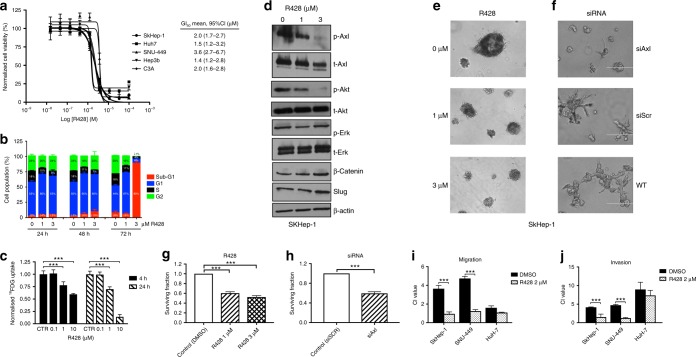
Inhibition of Axl induces phenotypic changes in Axl-overexpressing hepatocellular carcinoma (HCC) cell lines. **a** Sulphorhodamine-B (SRB) assays on a panel of immortalised HCC cell lines showing the dose-dependent growth inhibitory effect of R428 after 72-h incubation at day 2. **b** Flow cytometry analysis with propidium iodine staining demonstrating a dose and time-dependent sub-G1 cell cycle arrest following treatment with R428 at 1 and 3 μM for 24, 48 and 72 h. **c** ^18^FDG cell uptake study in SKHep1 cells following treatment with R428 for 4 and 24 h showing a significant and dose-dependent reduction in ^18^FDG uptake. Mean of *n* = 3 ± SD. **d** The effect of R428 on phosphorylated and total protein levels evaluated by Western blotting following 24-h incubation at 1 and 3 μM. Cells were treated with rhGas-6 (100 ng/mL) 10 min prior to lysis. R428 induces a dose-dependent de-phosphorylation of Axl at Tyr^702^ and Akt at Ser^473^. **e**, **f** Representative micrographs of matrigel colony formation assays demonstrating quantitative and phenotypical changes in the growth pattern of SKHep-1 following incremental doses of R428 (1 and 3 μM) (**e**) and transient Axl-specific shRNA, which specifically induces the loss of an invasive growth pattern compared to controls 14 days after seeding (**f**). The white line indicates a distance of 400 μm in panel (**e**) and 100 μm in panel (**f**). Representative replicates of at least 3 experiments are shown. **g**, **h** Quantification of colony formation assays presented as a fraction of surviving cells normalised to controls. **i**, **j** Effect of R428 on migration and invasion of cells with high (SKHep-1, SNU-449) versus low Axl expression levels (HuH7) using the xCELLigence assay. Motility and invasion through matrigel is reported at 24 and 48 h using normalised impedance values (cell index, CI). ***p < 0.001

We tested the cytotoxic potential of Axl inhibition using R428, an Axl-specific small molecule inhibitor with proven anti-tumour efficacy in vitro and in vivo.^34^ Using SRB assays, we confirmed R428 to exert growth inhibitory effects in the micromolar range across a panel of HCC cell lines after 72 h of continuous exposure to the drug (Fig. 2a).

We elected to further characterise the biologic functions of Axl using SKHep-1 (mesenchymal Axl-overexpressing cell line) and Huh-7 cells (epithelial, Axl-negative cell line). Flow cytometry confirmed R428 to induce G1-cycle arrest, consistent with the engagement of apoptosis in the Axl-overexpressing SKHep-1 cell line (Fig. 2b).

We subsequently tested whether R428-mediated Axl inhibition might affect downstream cell metabolism using ^18^F-FDG. Consistent with our hypothesis, we confirmed a dose-dependent decrease in ^18^F-FDG uptake following 6 and 24 h of treatment with R428 (Fig. 2c). To investigate the molecular pathways involved in such response, we focused on PI3K/Akt and Erk/MAPK given their renowned role as principal intracellular effectors of Axl biological functions.^13^ By Western blot, we confirmed R428 to induce a dose-dependent de-phosphorylation of Akt at the Ser^473^ residue with no effect on Erk1/2 phosphorylation, readout of MAPK cascade activation (Fig. 2d).

We used matrigel colony-formation assays to evaluate phenotypical changes in the growth pattern of SKhep-1 cells following Axl inhibition and demonstrated R428 to impair colony formation in the micromolar range as shown in Fig. 2e, f. Silencing of Axl using target-specific shRNAs (Supplementary Figure 3) induced phenotypic changes in the pattern of growth of SKHep-1 cells as well as reduction in the number of colonies to confirm the specificity of the growth inhibitory effects shown for R428 (Fig. 2f, h). Following silencing of Axl by shRNAs, we observed morphologic changes in the pattern of growth of SKHep-1 cells which were not shared with R428-treated counterparts, where cells proliferated in progressively smaller discoid-shaped structures, characterised by higher cell density. Following Axl-specific shRNA inhibition SKHep-1 cells developed an inferior tendency to a branched morphogenesis of in vitro colonies in favour of the evolution of small, circular structures.

### Role of Axl and EMT in the acquisition of adaptive resistance to sorafenib

**Fig. 3:**
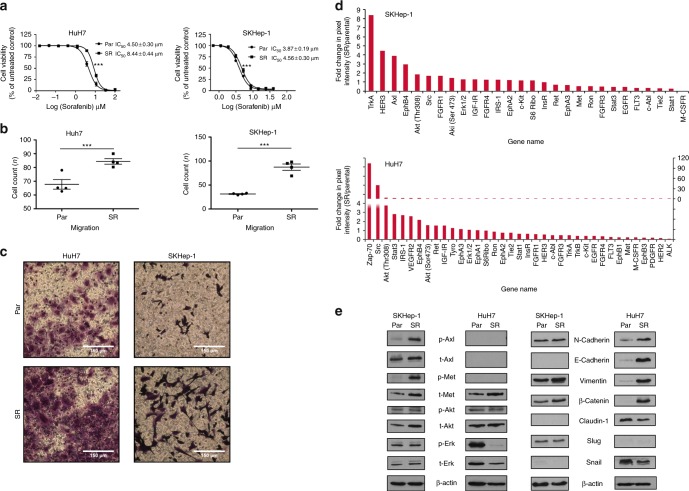
The relationship between epithelial-to-mesenchymal transition (EMT) and Axl activation in the development of acquired sorafenib resistance in immortalised hepatocellular carcinoma (HCC) cell lines. **a** Sulphorhodamine-B (SRB) assays demonstrating a significant shift in the GI_50_ following chronic sorafenib incubation in HuH7SR and SKHep-1SR compared to their parental counterparts. **b**, **c** Migration assays demonstrating a significant increase in cell motility following the acquisition of sorafenib-resistance in HuH7 and SKHep-1 cells. **d** Antibody array experiments demostrating the differential activation of 28 RTK and 11 signalling nodes in sorafenib-resistant cell clones compared with naive counterparts. A 4-fold increase in Axl phosphorylation is noted in SKHep-1SR cells compared to their sorafenib-naive counterparts. **e** Analysis of the expression of Axl as well as key EMT-related proteins and Axl-related signalling nodes demonstrating a significant increase in Axl and Met phosphorylation in SKHep-1SR cells and significant overexpression of E-cadherin, Vimentin, β-Catenin together with Claudin repression in HuH7SR cells suggesting differential activation of EMT across cell lines in the context of sorafenib resistance. Representative replicates of at least 3 experiments are shown. ***p < 0.001

We derived sorafenib-resistant cells clones (HuH7-SR and SKHep-1-SR) from parental HuH7 and SKHep-1 cell lines following chronic exposure to incremental concentrations of sorafenib as described before.^35^ Sorafenib-resistant cell clones were confirmed using SRB assays (Fig. 3a). HuH7-SR and SKHep-1-SR displayed increased migration and invasion capacity compared to sorafenib-naive counterparts (Fig. 3b, c).

To comprehensively characterise the molecular pathways underlying sorafenib-resistance, we performed antibody-array screening to simultaneously detect the phosphorylation of 28 RTKs and 11 downstream signalling nodes in parental and resistant HCC cell clones. As shown in Fig. 3d, we demonstrate differential activation of intracellular pathways in the two cell lines.

We confirmed the activation of the PI3K/Akt pathway, Src and EphB4 as a shared trait across the two studied cell lines. Conversely, Zap-70 and STAT-3 were typically up-regulated in the primarily epithelial HuH7 cell line, whereas TrkA, Her-3 and Axl emerged as significantly hyperphosphorylated in sorafenib resistant, primarily mesenchymal SKHep-1 cell clones. Validation of antibody arrays by immunoblot confirmed Axl hyperphosphorylation SKHep-1 but not in HuH-7 cells, where we found a significant up-regulation of EMT-related proteins including N-Cadherin, E-Cadherin, Vimentin and β-catenin (Fig. 3e).

### Inhibition of Axl modulates cell motility, invasion and sensitivity to sorafenib in resistant cell clones

**Fig. 4:**
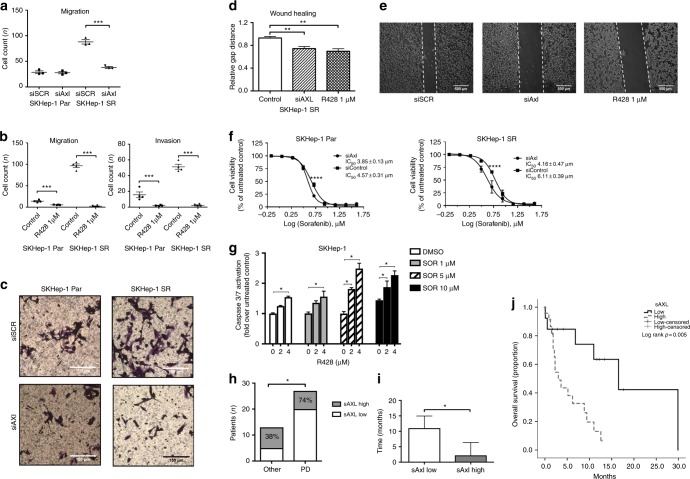
Axl modulates the motility and invasive capacity of immortalised hepatocellular carcinoma (HCC) cells and determines sensitivity to sorafenib in immortalised cell lines and in patients with HCC. **a**, **b** Inhibitory effect of shRNA-mediated Axl silencing on SKHep-1 and SKHep-1SR cell motility and invasion capacity. **c** Inhibitory effect of R428 on SKHep-1 and SKHep-1SR cell motility and invasion. **d**, **e** Wound healing assays confirming shRNA and R428-mediated repression of cell motility secondary to Axl inhibition. **f** Inhibition of Axl by shRNA augments the sensitivity of SKHep-1 and SKHep-1SR cells to sorafenib. **g** Incremental effect of R428 on sorafenib-induced apoptosis of SKHep-1 cells as measured by Caspase 3/7 activation ratios against controls. **h** The relationship between pre-sorafenib serum sAxl levels and cause of treatment discontinuation in patients receiving sorafenib for HCC (*n* = 40). **i** Patients with elevated sAxl levels had significantly shorter duration of sorafenib therapy with earlier discontinuation compared to patients with lower sAxl levels at baseline. **j** Kaplan–Meier curves showing the effect of high baseline sAxl levels in predicting for worse overall survival in patients with HCC undergoing sorafenib therapy

We further characterised the biologic significance of Axl activation in sorafenib-resistant cell clones. Using shRNAs we demonstrated a significant Axl-dependent decrease in motility and invasion capacity in SKHep-1-SR cells (Fig. 4a, b). Axl downregulation by siRNA silencing or R428 also impaired cell migration in wound healing assays (Fig. 4c–e). Using SRB assays we demonstrated that SKHep-1 cells and their sorafenib-resistant counterparts displayed increased sensitivity to sorafenib following siRNA-mediated Axl downregulation (Fig. 4f). To inform the clinical development of Axl inhibitors in HCC, we postulated whether the addition of R428 to sorafenib in SKHep-1 cells might be used to delay/prevent acquired sorafenib resistance by enhancing cell cytotoxicity in sorafenib-naive cells. First, we evaluated whether R428 could influence sorafenib-mediated apoptosis using Caspase 3/7 activation as a readout (Caspase-Glo Assay, Promega, Madison, WI, USA). We found an incremental increase in apoptosis by a combination of increasing doses of R428 with sorafenib (Fig. 4g).

We validated this finding using the Chou–Talalay method in formal combination cytotoxicity studies on SKHep-1 cells, using SRB assays as a readout^36^ where we confirmed that the addition of R428 exerted additive anti-proliferative effects at ED50, with CI value of 0.99 and synergistic effects at ED75 and ED90, as shown by CI values of 0.74 and 0.57, respectively (Supplementary Figure 4).

### The clinico-pathologic role of soluble Axl levels in patients with advanced HCC treated with sorafenib

To validate our in vitro findings, we measured the concentration of soluble Axl (sAxl) in serum samples from 40 patients presenting with HCC and treated with sorafenib between 2011 and 2016 at Imperial College, London as per international guidelines^37^ until disease progression or unacceptable toxicity. Patient characteristics are shown in Table 1.

**Table 1:** Clinical features of HCC patients treated with sorafenib (*n* = 40)

Characteristic	*N* = 40 (%)
Age in years, median (range)	63 (45–85)
Gender	
Male	32 (80)
Female	8 (20)
Aetiology of liver disease	
Viral	21 (53)
Non-viral	19 (47)
AFP	
<400 ng/mL	18 (45)
>400 ng/mL	17 (42)
Missing	5 (13)
Child-Turcotte Pugh Class	
A	22 (54)
B	18 (46)
Maximum diameter of largest lesion (cm)	
Median (range)	6 (2–15)
Extrahepatic spread	
Absent	25 (63)
Present	15 (37)
Portal vein involvement	
Absent	27 (67)
Present	13 (33)
BCLC stage at sorafenib initiation	
A–B	16 (40)
C	24 (60)
CLIP score	
0–1	19 (47)
>1	21 (53)
Number of prior treatments lines	
None	24 (65)
≥1	16 (35)
Sorafenib discontinuation	
Progressive disease	27 (67)
Toxicity	6 (15)
Others	3 (8)
Ongoing	4 (10)

The median duration of sorafenib treatment was 3 months (range 2 weeks to 30 months), with 27 patients (60%) developing disease progression during the study period. Median sAxl levels at the commencement of sorafenib were 2682 pg/mL (range 78–3000 pg/mL). Serum sAxl levels were higher in patients who discontinued sorafenib due to radiologically proven disease progression (*n* = 20/27, 74% versus *n* = 3/13, 38% for other causes, Fisher’s exact test *p* = 0.04, Fig. 4h). Patients with higher sAxl levels prior to sorafenib (*n* = 25) had significantly lower duration of sorafenib therapy compared to patients with lower sAxl levels (*n* = 15, median duration 2.2 months, 95%CI 2.3–5.7 for Axl-high versus 11 months, 95%CI 4.7–15, Mann–Whitney *p* = 0.03, Fig. 4i).

The median OS was 12.1 months (95%CI 1.4–22.7) for the whole patient cohort and worse in patients with higher sAxl levels with a median OS of 3 months (95%CI: 1.2–4.8) versus 16.5 months (95%CI 4.6–28.4) in patients with lower sAxl levels (Log-rank *p* = 0.02, Fig. 4j).

## Discussion

The paucity of systemic treatments for HCC makes the management of patients who progress after standard therapies a particularly challenging clinical scenario.^38^ Since the approval of sorafenib in 2007, pre-clinical studies have contributed to elucidate the role of an increasing number of signalling pathways including EGFR/Her-3,^39^ insulin-like growth factor (IGF),^40^ fibroblast growth factors (FGF)^41^ and many others as molecular traits that limit the efficacy of sorafenib, mostly by induction of proliferation through up-regulation of intracellular second messengers including the PI3K/Akt and Erk/MAPK pathways.^24^ Despite being built on a solid pre-clinical rationale, selective targeting of most of these pathways has unfortunately proven ineffective in clinical trials.^42^ In parallel, the marginal survival benefit observed with the second-line use of regorafenib, a TKI with high structural homology and similar pharmacodynamic properties of sorafenib, highlights the acute need to further characterise the mechanisms underlying acquired sorafenib resistance in HCC.^43^

Compelling evidence suggests that Axl tyrosine kinase is a molecular determinant in the progression of a number of malignancies including HCC,^19^ being implicated in cell proliferation, motility and metastasis through modulation of EMT,^44^ a key pathway involved in drug resistance.^22^

In our study, we confirm Axl overexpression to be a common molecular event in HCC. Analysis of the TCGA dataset revealed a significant positive correlation between Axl, Vimentin and Slug mRNA, a zinc-finger transcription factor implicated in E-cadherin repression during EMT^45^ and central to the promotion of Axl-mediated invasion in HCC cells.^17^

We confirmed Axl protein expression in human tissue, where strong Axl immunolabeling was seen in 60% of the examined primary HCC samples, with a positive gradient from histologically normal, cirrhotic controls to cancerous tissue that suggests a role for Axl in the acquisition of the transformed phenotype. Interestingly, Axl was overexpressed in a subset of advanced cases and matched metastatic deposits, a finding that further substantiates the involvement of this RTK in the extra-hepatic progression of liver cancer.

Mechanistically, we explored these findings further using two molecularly distinct cell lines: SKHep-1, an HCC cell line with mesenchymal phenotype derived from metastatic HCC^46^ and HuH-7, a fully epithelial cell line derived from a primary HCC.^47^

Using a number of readouts of cell proliferation, motility and invasion capacity, we have shown that therapeutic modulation of Axl by genetic down-regulation as well as pharmacologic inhibition by R428, a well-characterised TKI with selective Axl-inhibitory properties,^34^ affects a wide range of hallmarks underlying the progression of HCC.

We found that treatment with R428 resulted in cell cytotoxicity across an array of HCC cell lines with GI_50_ values ranging within the low-micromolar range and confirmed this effect to be secondary to drug-induced suppression of cell proliferation and promotion of apoptosis. Upon inhibition of Axl tyrosine kinase activity, R428 preferentially reduced the activation of PI3K/Akt in a dose-dependent manner, as shown in other solid tumours.^34,48^ Some of the downstream effects of R428 on the selected in vitro readouts of HCC tumorigenesis were evident at higher exposures (2–3 μM), which were well within the clinically achievable dose range of 1–4 μM observed in other tumour types.^49^

In our experiments, we did not find a significant difference in GI_50_ values for R428 based on Axl protein expression, suggesting a more complex relationship between drug exposure and its downstream antiproliferative effects, some of which may be due to ‘off target’ activity. This is a point of greater consequence in the clinical development of Axl-directed therapies for which no response predictor exists. Interestingly, this finding mirrors the clinical experience of c-Met inhibitors in HCC,^50^ where the lack of association between target expression and therapeutic efficacy suggests the need for ongoing research efforts to be addressed at the discovery of biomarkers that can predict benefit from EMT-targeting agents.

In light of the increasing body of evidence suggesting that Axl is a master regulator of resistance to TKIs,^22^ we hypothesised that Axl phosphorylation might be mechanistically involved in the development of resistance to sorafenib. To verify this, we first adopted an unbiased approach by evaluating multiple signalling networks involved in shaping the resistant phenotype. Interestingly, our analysis validated renowned hallmarks of sorafenib resistance including Her-3, FGFRs, IGFR, Akt and STAT-3, a finding that validates our in vitro model of resistance.^24^

Subsequently, we verified that sorafenib-resistant clones displayed increased migratory and invasive potential as a unifying trait across cell lines. Interestingly, this phenotype correlated with a different protein-signalling profile: Axl-phosphorylation was restricted to cells lines with baseline activation of EMT and not to those with a more epithelial phenotype, where the increased migratory potential was associated with up-regulation of Vimentin, N and E-cadherin and down-regulation of Claudin-1.

The differential activation of EMT we observed, with HuH7-SR cells exhibiting an intermediate epithelial–mesenchymal phenotype that is Axl-independent compared to SKHep-1 cells, is translationally relevant as it suggests Axl signalling to be specifically enriched in HCC cells that have undergone full mesenchymal differentiation. This highlights the existence of a subset of patients in whom Axl inhibition might serve as a strategy to circumventing the acquisition of sorafenib-resistance.^51^

With intra-tumour heterogeneity having been described as a mechanism to explain the differential activation of molecular drivers of HCC,^52^ it is perhaps unsurprising that the mechanisms of resistance to therapy might not be shared amongst cells of different lineage.^53^ It has been shown that patients displaying extra-hepatic HCC progression post-sorafenib carry a significantly poorer prognosis,^54^ an observation that finds a potential mechanistic explanation in the increased migratory and invasive potential that we documented in our in vitro model of resistance.

We found that the survival of patients treated with sorafenib was inversely proportional to plasma Axl concentration, further substantiating the pathophysiologic importance of Axl in affecting clinically meaningful outcomes in HCC. Interestingly, increased cell surface availability of Axl through reduced receptor proteolysis and shedding has been described as a resistance mechanism to MAPK-signalling inhibitors and might account for the worse survival figures seen during sorafenib treatment.^55^ Our results suggest that Axl is a putative therapeutic target expressed in primary and metastatic HCC and that its inhibition influences the sensitivity of HCC cells to sorafenib and reverts the hypermotile phenotype that accompanies adaptive drug resistance.^23^

Given the broad spectrum of its downstream molecular targets, it is perhaps unsurprising to note the pleiotropic effects of sorafenib in inducing the compensatory activation of a number of molecular pathways as well as EMT, some of which, including Trk-A and EphB4 should be comprehensively characterised in future studies. The breadth of genetic and post-translational modifications induced by sorafenib and the lack of oncogenic addiction in HCC makes it unlikely for a single molecular pathway to exert gatekeeper effects in the development of acquired resistance.

In our model, the finding that chronic treatment with sorafenib led to a <2-fold shift in GI_50_ values based on formal cytotoxicity assays suggests this phenotype to be potentially reversible and substantially different from stable acquired drug-resistance phenotypes seen in other oncologic indications, where more profound shifts in GI_50_ values are often determined by the acquisition of secondary mutations.^56^ Interestingly, these findings mirror clinical experience in HCC, where sorafenib largely induces cytostatic effects and progression occurs after a variable period of disease stability.^57^

Nevertheless, our study suggests Axl as a promising therapeutic target in HCC and qualifies R428 as a biologically active compound with antiproliferative effects within the low micromolar range in a wide array of immortalised HCC cell lines. The incremental effects of R428 on sorafenib-induced growth inhibitory and pro-apoptotic potential are promising features for the development of Axl-inhibitors in the first-line treatment of advanced HCC aiming to prolong progression-free survival before patients face untreatable progression due to liver dysfunction or symptomatic progression. Axl-induced modulation of motility and invasion is suggestive of a potential anti-metastatic role of R428, a finding that replicates previous evidence in other tumours^34^ and warrants further investigation clinical studies. Our results suggest Axl to be involved in the metastatic progression and therapeutic resistance, two major clinical determinants of mortality in HCC. Consideration should be given to advanced HCC as a promising therapeutic indication for the development of Axl inhibitors. The widening clinical experience with R428 (bemcentinib, BGB324) as a monotherapy or in combination with other small molecule inhibitors suggests this molecule to be an ideally suited candidate for further clinical evaluation.

## Supplementary information

Supplementary Data

## Data Availability

Primary research data are presented in a summative fashion in the manuscript. No publicly available dataset has been generated as part of this work.
