# Supplementary material for: Integrated analysis of multiple receptor tyrosine kinases identifies Axl as a therapeutic target and mediator of resistance to sorafenib in hepatocellular carcinoma
Source: Br J Cancer. 2019 Feb 15;120(5):512–21. doi: 10.1038/s41416-018-0373-6 (PMC6461770; doi:10.1038/s41416-018-0373-6)
Supplement: Supplementary file 1 — Supplementary Data [file 41416_2018_373_MOESM1_ESM.docx]

**Integrated analysis of multiple receptor tyrosine kinases identifies Axl as a therapeutic target and mediator of resistance to sorafenib in hepatocellular carcinoma.**

Pinato D.J. et al.

**Supplementary Materials and Methods.**

**Cell Culture.** Cell lines were grown in either Dulbecco Modified Eagle’s Medium or Roswell Park Memorial Institute Medium as recommended by American Type Tissue Collection (ATCC) guidance. Media were supplemented with 1% penicillin/streptomycin and 10% (v/v) fetal calf serum (Sigma Aldrich, St. Louis, MO, USA) and grown at 37 °C in 5% CO_2_ atmosphere.

**Immunoblot.** The following antibodies were used: Axl (Cell Signaling Technology Inc., Danvers, MA, USA); N-Cadherin (D4R1H), E-Cadherin (24E10), Vimentin (D21H3), β-Catenin (D10A8), Claudin-1 (D5H1D), Slug (C19G7), Snail (C15D3), p44/42 MAP kinase (137F5) and phospho-p44/42 MAP kinase (Thr202/Tyr204, D13.14.4E), pan-AKT (C67E7) and phospho-AKT (Ser473, D9E) (all from Cell Signaling Technology Inc.), Gas-6 (AF885, R&D Systems, Minneapolis, MN, USA), phosphor-Axl (Tyr779, AF2228, R&D), β-actin (AbCam, Cambridge, UK).

**R428 treatment.** SKHep-1 cells were cultured in 6 well plates and treated with R428-supplemented media (1 μM, 3 μM) or DMSO control for 24 hours. To evaluate the effect of R428 in modulating Axl-related signaling we treated cells with recombinant human Gas-6 (R&D Systems) for 10 minutes before immediate lysis in RIPA buffer (Invitrogen, cat. nr 89900) supplemented with phosphatase and protease inhibitors (Thermo Scientific, cat nr. 78440) and downstream immunoblot analysis.

**Growth inhibitory assay.** Drug concentrations that inhibited 50% of cell growth (GI_50_) were determined using sulforhodamine B assays, following optimization of cell densities per each individual cell line. Optical densities were measured at 540 nm with a Multiskan EX photometer using the Ascent software (version 2.6; Thermo Labsystems, Altrincham, UK). Growth inhibition curves were plotted as percentage of control cells and GI_50_ values were determined by GraphPad Prism version 7 software (GraphPad Software, San Diego, CA, USA) by fitting a sigmoidal curve with variable slope.

For combination studies, SKHep-1 cells were treated with a series of drug concentrations: sorafenib (0–100 μgM) and R428 (0–100 μM) alone or in a fixed concentration of R428 (3 μM). Cells were incubated with the drugs at the indicated concentrations for 72 hours. The ED50, 75 and 90 values was defined as the concentration needed for a 50%, 75% and 90% reduction in cell viability. Dose–effect analyses and ED50, ED75 and ED90 calculations were performed using Compusyn software 1.0 (Combosyn Inc., Paramus, NJ, USA). The effects of different drug combinations were determined as previously described by Chou-Talalay, with a Combination Index (CI) being presented to derive a quantitative definition for additive effect (CI = 1), synergism (CI < 1), and antagonism (CI > 1) in the sorafenib/R428 combination.

**Apopotosis Assays.** We studied the effect of R428 in influencing sorafenib-induced apoptosis by combination treatment. Cells were plated and treated with DMSO (control) or sorafenib and R428 at pre-defined concentrations (1, 5 and 10 μM for sorafenib and 2 and 4 μM for R428). Caspase 3/7 activation was utilized as a readout by using the Caspase-Glo Assay (Promega, Madison, WI, USA) in accordance to the manufacturer’s instructions. Caspase 3/7 activation was measured as a ratio compared to untreated controls. P-values generated from the multiple comparisons of Caspase 3/7 ratios across the different concentration points were adjusted using the Dunnet’s multiple comparisons test.

**Generation of an isogeneic collection of primary and metastatic HCC samples.** We generated a series of matched primary and metastatic HCC using post-mortem (PM) specimens retrieved through the Imperial College Tissue Bank. We have preliminary screened the reports of 12.580 PM examinations performed between 1970 and 2005 adopting stringent tissue quality criteria: 1) confirmed histopathologic diagnosis of metastatic HCC; 2) lack of significant autolytic degeneration; 3) complete PM dissection of all the organs; 4) post-mortem interval <24 hours from death.

Review of PM epicrisis and medical notes allowed reconstruction of ante-mortem clinico-pathologic features. Each patient underwent sampling of primary and secondary lesions at the time of macroscopic dissection, followed by routine formalin fixation and paraffin embedding (FFPE).

All cases were preliminarily evaluated on freshly cut hematoxylin & eosin (H&E) slides by a consultant histopathologist (FAM) to confirm histotype and tissue preservation. Preliminary staining of tissue sections with the pan-cytokeratin marker MNF116 and the hepatocyte-specific Hep-par1 marker was utilized to ascertain adequate tissue preservation for immunohistochemical assays as performed by our group in previous studies^1^.

**Immunofluorescence.** Wild type SKHep-1 cells (3 × 10^4^) were seeded into 4-well chamber slides (BD Biosciences) alongside clones transfected with Axl-specific shRNA and non-targeted sequences as controls. Cells were cultured overnight and washed 2 × 5 minutes with PBS and fixed with 4% formaldehyde (Sigma-Aldrich) in PBS for 15 minutes at room temperature. Cells were washed 3 × 5 minutes with PBS and permeabilized with 100 μL 0.1% Triton X-100 in PBS for 10 minutes at room temperature. Following permeabilization, cells were washed again 3 × 5 minutes with PBS at room temperature and blocked with 1% BSA / 0.1% Triton in PBS (PBST-BSA) for 1 h at room temperature. Cells were incubated with anti-Axl primary antibody (Cell Signaling Technology) at a dilution of 1:60 in 100 μL/well PBST- BSA overnight at 4°C. The next day, cells were washed 3 × 10 min PBS and incubated with secondary Alexa fluor 594 goat anti-mouse IgG antibody (AbCam, Cat. Nr: ab150080) at a dilution of 1:400 in 100 μL PBST-BSA for 1 h at room temperature in the dark. Cells were washed 3 × 10 minutes with PBS and coverslips mounted using ProLong Gold Antifade Reagent containing DAPI (Invitrogen, Cat. Nr: P-36931). Images were acquired on an Olympus BX51 microscope and DP controller software version 2.1.

**Real Time Cell Analysis**. RTCA for migration and invasion in response to R428 was performed using the xCELLigence platform (Acea Bioscience, San Diego, CA, USA). For the purpose, we employed CIM-16-well plates which are endowed with interdigi­tated gold microelectrodes on bottom side of a filter membrane interposed between a lower and an upper compartment. The lower chambers were filled with serum-free medium (control) or growth medium (10% FBS). Viable cells (2x10^4^ cells/well) were seeded on filters in serum-free medium supplemented with either DMSO or R428. For invasion studies, upper chambers were coated with 2% matrigel. Impedance changes detected by the microelectrodes are proportional to the number of migrating cells and are expressed as cell index (CI). Migration was monitored in real-time for 48 h, with landmark timepoints being utilized for analysis (24 and 48h for migration and invasion respectively). Each experiment was performed three times in quadruplicate.

**Supplementary Data.**

| **ID** | **Maximum Primary Tumor Diameter** | **Age** | **Gender** | **Focality** | **Etiology of Liver Disease** | **Number of Metastatic Sites** | **Portal Vein Thrombosis** | **Sites of Metastasis** | **Axl IHS Primary** | **Axl IHS Metastasis** |
| --- | --- | --- | --- | --- | --- | --- | --- | --- | --- | --- |
| 1 | 7.5 | 70 | M | Multifocal | Unknown | 1 | No | Regional Lymphnodes | 100 | 100 |
| 2 | 6 | 60 | M | Multifocal | Hemochromatosis, Alcohol Excess | 1 | No | Lung  Right Adrenal | 70 | 120 (Lung)  70  (Right Adrenal) |
| 3 | 5 | 74 | F | Multifocal | Unknown | 2 | Yes | Regional Lymphnodes  Pancreas* | 180 | 100  (Regional Lymphnodes) |
| 4 | 10 | 44 | M | Multifocal | Unknown | 2 | Yes | Spleen | 200 | 100 |
| 5 | 12 | 79 | M | Unifocal | Hemochromatosis | 3 | No | Multifocal lung,  Right Adrenal | 100 | 100 (Lung)  70  (Right Adrenal) |

**Supplementary Table 1.** Immunohistochemical expression of Axl in isogeneic primary and metastatic deposits of hepatocellular carcinoma: clinicopathologic features of the patient cohort.

* Not sampled due to insufficient quantity.

**Supplementary Figure 1.** The relationship between Gas-6 and Axl mRNA expression from the CCLE (**Panel A**) and TCGA (**Panel B**) RNA-seq datasets.

**
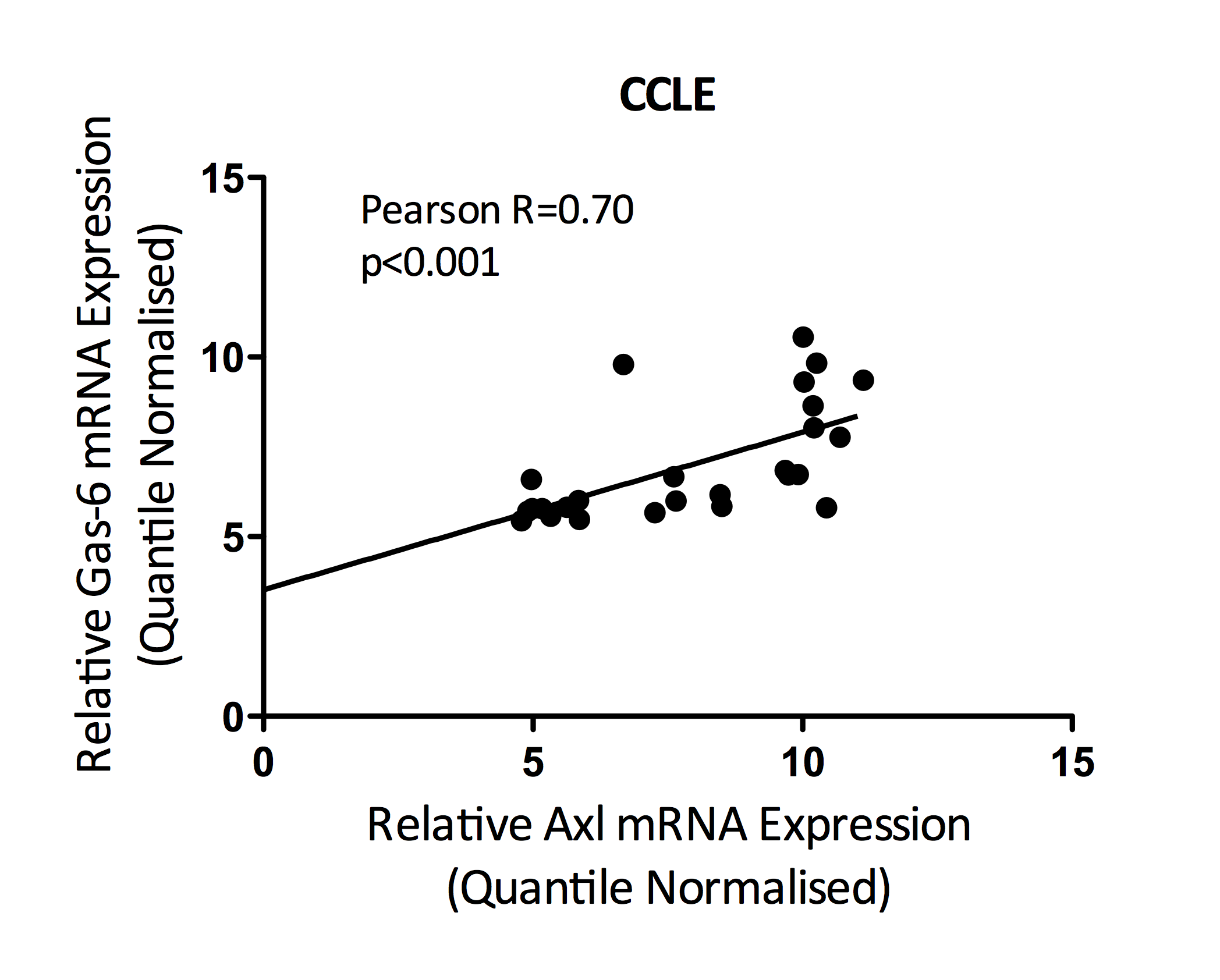
**

**A.**

**
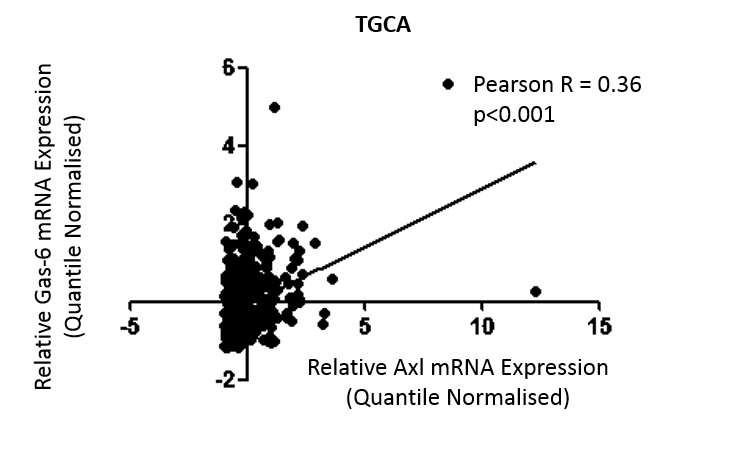
B.**

**Supplementary Figure 2.** The relationship between Axl expression and Akt phosphorylation by Western Blotting in a panel of HCC cell lines. HCT-116 cell lysates were used as controls.

**
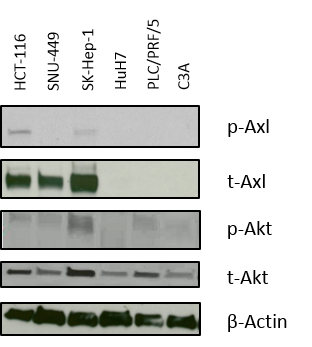
**

**Supplementary Figure 3.** Immunofluorescence microscopy experiments illustrating efficient Axl knockdown in target-specific siRNA SKHep-1 transfectants (**Panel A**) in comparison with clones transfected with non-target siRNA sequences (**Panel B**) and SKHep-1 wild type clones (**Panel C**).


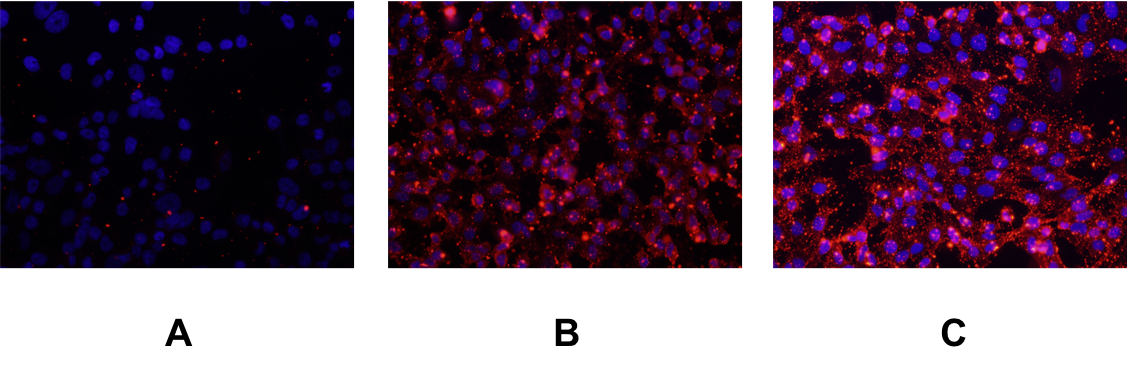


**Supplementary Figure 4.** Combination study of sorafenib and R428 using SRB assays as readout for the anti-proliferative effect of the combination. **Panel A** Logarithmic combination index (CI) plot illustrating the Log(CI) value plotted against effect (Fa) of the combination. **Panel B** shows isobolograms for 50%, 75% and 90% inhibition.

**A**


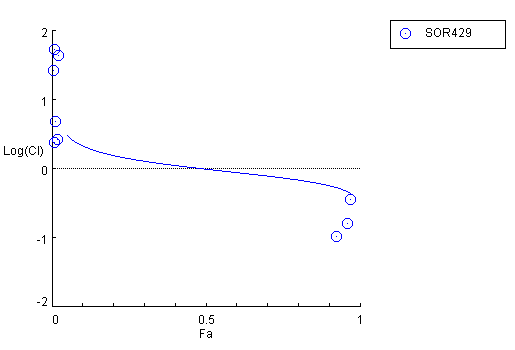


**B**


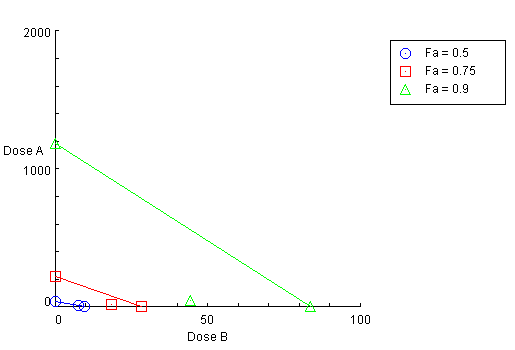


**Supplementary References.**

1. Pinato DJ, Shiner RJ, White SDT, et al. Intra-tumoral heterogeneity in the expression of programmed-death (PD) ligands in isogeneic primary and metastatic lung cancer: implications for immunotherapy. OncoImmunology. 2016:00-.
